# Medical-Legal Partnerships for Women With Co-Occurring Intimate Partner Violence and Opioid Use Disorder: An Exploratory Study

**DOI:** 10.1177/10778012251347622

**Published:** 2025-06-09

**Authors:** Penelope Morrison, Katherine McLean, Richard Wentling, Elizabeth Schachte, Bobur Rakhmatullaev

**Affiliations:** 1 43326 Penn State New Kensington, New Kensington, PA, USA; 2 52497 Penn State Greater Allegheny, McKeesport, PA, USA; 3 43312 Penn State Behrend, Erie, PA, USA

**Keywords:** intimate partner violence, opioid use disorder, social determinants of health, coordinated care, medical-legal partnerships

## Abstract

We interviewed 40 individuals working across service domains (e.g., healthcare, criminal justice) to understand their perspectives on the barriers and facilitators to employing a medical-legal partnership (MLP) in settings which serve women with co-occurring intimate partner violence and opioid use disorder (co-IPV/OUD). Providers cited “buy-in” from all stakeholders, establishing shared goals, objectives, and practices across stakeholders, training on IPV and OUD, and/or the need for additional staff, and funding as key considerations for MLPs in this context. Our study finds that MLPs represent a viable and promising potential pathway to providing more co-located or coordinated care for women with co-IPV/OUD.

## Introduction

Intimate partner violence (IPV) and opioid use disorder (OUD) remain significant threats to the health of women and their families in the United States. Rates of IPV and opioid misuse have risen in recent years, and both are now considered leading causes of death for reproductive-aged women (National Center for Health Statistics, 2023; Spencer et al., 2023; Thompson & Tapp, 2022). Furthermore, research has shown that IPV is often associated with substance use disorders (SUDs), including OUD—meaning that women who experience IPV are more likely to be diagnosed with a substance use disorder and/or vice versa, that women with SUD have a greater risk for IPV victimization (Mehr et al., 2023; Ogden et al., 2022; Stone & Rothman, 2019). Rates of co-occurring IPV and OUD (co-IPV/OUD) are understudied and arguably there are methodological issues that make it difficult to measure the exact number of women experiencing both (Stone & Rothman, 2019). Additionally, given the high level of criminogenic risk factors that are associated with OUD and the high level of stigma that both OUD and IPV carry, it is also highly likely that co-IPV/OUD is significantly unreported (Morse et al., 2022). However, some studies have found estimates as high 94% lifetime physical IPV victimization among women who have ever used nonprescription opioids (Frye et al., 2001), while others have found that 46–50% of IPV victims have a history of prescription opioid use (Stene et al., 2012). Furthermore, one systematic review by Stone and Rothman (2019) synthesized prevalence rates of co-IPV/OUD and concluded that there is “little doubt that it is more likely than not that a woman in methadone treatment has experienced IPV victimization in her lifetime and is very probable that she experienced it in the past year” (Stone & Rothman, 2019, p. 225). Thus, co-IPV/OUD is clearly an emergent issue that warrants attention—and, more specifically, research is needed to understand how to best address these issues concurrently.

Research suggests that the co-occurring nature of IPV and OUD presents a unique set of consequences for women experiencing both issues (Hill et al., 2024; Pallatino et al., 2021; Phillips et al., 2021; Stone et al., 2021). Often women experiencing co-occurring IPV/OUD have a high level of unmet needs that make it difficult for them to seek help and/or engage in treatment (e.g., Allen et al., 2024; Hooker et al., 2020). Studies on IPV and OUD have shown that women experiencing these issues are more likely to experience resource scarcity, employment instability, or to be low-income (Allen et al., 2024; Hooker et al., 2020; Rose-Jacobs et al., 2019). They often also have a high need for assistance from financial aid programs and/or supplemental programs such as TANIF or SNAP, have limited or no health insurance, experience housing insecurity, transportation limitations, and difficulties securing resources or care for their children (Allen et al., 2024; Huhn & Dunn, 2020; Rose-Jacobs et al., 2019; Stone et al., 2021). Additionally, women experiencing IPV and OUD have a high level of unmet health needs and often present in clinical settings with mental health concerns (e.g., depression, anxiety, and PTSD) and/or physical health problems (e.g., chronic pain, sexual and reproductive health needs) that have not been addressed (Allen et al., 2024; Huhn & Dunn, 2020; Mehr et al., 2023). Research also shows that this population can encounter heighted barriers to engaging in and receiving care, including, but not limited to, fear of partner retaliation, financial abuse or the partners’ control of money and other resources (e.g., insurance cards), treatment sabotage, and substance use coercion (Hill et al., 2024; Khan et al., 2022; Morrison et al. 2022; Pallatino et al., 2021; Phillips et al., 2021). Women experiencing IPV and OUD also sometimes face discrimination or negative treatment by providers as barriers to care (Allen et al., 2024; Huhn & Dunn, 2020; Khan et al., 2022; Phillips et al., 2021). In fact, co-IPV/OUD presents amplified stigma due to the presence of both conditions, which can prohibit women from seeking care in the first place (Hill et al., 2024; Huhn & Dunn, 2020; Morrison et al., 2022; Stone et al., 2021). Additionally, OUD is often associated with other criminogenic risk factors (Morse et al., 2022); thus, women with co-IPV/OUD may fear the socio-judicial consequences that can result from initiating contact service providers, including fear of the police or reluctance to have law enforcement involved, fear of being arrested or jailed, or other legal consequences (e.g., losing custody of children, either to the abuser or system, child, youth, and family services involvement, eviction from their home, etc.) all may present barriers to women seeking care for these conditions (Morrison et al., 2022; Phillips et al., 2021).

Co-IPV/OUD also presents a set of challenges for service providers who work with this population and who are tasked with intervention, treatment, advocacy, and care (Morrison et al., 2022). Despite the recognition that IPV and OUD are often co-occurring, and present with heightened needs and greater barriers to care, these conditions are still largely treated in silos and forging a path to co-located or coordinated care for women experiencing both has historically been difficult (Bennett & Lawson, 1994). A recent study by Morrison et al. (2022) found that individuals who work with women experiencing IPV and OUD reported both service provision level and structural barriers to treating co-IPV/OUD holistically. Issues such as a lack of screening for both IPV and OUD across service provision contexts, provider discomfort with IPV or OUD, and provider bias often prohibited providers from addressing IPV and OUD together. Furthermore, a lack of options for in-patient help for women experiencing co-IPV/OUD, difficulties working and communicating across service provision agencies, and limited funding options for co-located or coordinated care made it difficult to develop coordinated care models (Morrison et al., 2022). Nonetheless, the need for co-located or coordinated care for this population is evident (Morrison et al., 2023; Stone & Rothman, 2019) and studies of women experiencing co-IPV/OUD have found that they too report a strong desire for treatment models which are integrated and can provide a higher level of coordinated care (Hill et al., 2024; Phillips et al., 2021; Stone et al., 2021). Thus, figuring out new and innovative ways to address co-IPV/OUD, integrate care, and provide services that can help women overcome barriers to care for these issues is important.

### Medical-Legal Partnerships as Potential Pathway to Care for Co-IPV/OUD

Medical-legal partnerships (MLPs) represent one potential pathway to bridging the gaps in care for women experiencing co-occurring IPV/OUD and are potentially an important inroad into developing models of coordinated care for this population. MLPs were first established in the 1990s by Barry Zuckerman at Boston Medical Center; since this time, they have become increasingly popular and today 450 partnerships exist across 49 states and the District of Columbia, mostly housed in hospitals and community health clinics across the country (National Center for Medical-Legal Partnership, 2024). At their core, MLPs seek to incorporate legal aid or legal representation in clinical settings which serve highly vulnerable populations of patients (Tobin Tyler, 2019; Tobin-Tyler & Teitelbaum, 2019; Welch et al., 2021). The model is based on the premise that the roots of health disparities can be found in an array of unmet social needs (e.g., poor quality housing, food insecurity, employment instability, lack of personal safety, etc.) which tend to predominantly impact low-income individuals, and negatively impact health, leading to disease or other adverse health outcomes (Girard et al., 2021; Martinez et al., 2017; Welch et al., 2021). These unmet needs are often referred to as the social determinants of health (SDOH) and research suggests that addressing social needs is just as important to health and health equity as providing medical interventions (Chelak & Chakole, 2023). However, healthcare systems often are ill equipped to address the SDOH, many of which may be associated with underlying unaddressed legal needs—needs which, if remedied, may in turn alleviate poor health and/or prevent it from occurring in the first place. In short, MLPs view unmet legal needs as “health-related social problem[s] that … [are] better addressed through legal assistance than traditional medical care” (Beeson et al., 2013, p. 2). Thus, while the goals of MLPs vary slightly from partnership to partnership, the main tenets of such programs are (a) to address the unmet socio-legal needs—and in particular, to address those needs in low-income, poorly resourced populations of individuals; (b) to improve overall health and wellbeing by remedying potentially health-harming legal needs (HHLNs); (c) and to advocate for policy and systems changes within healthcare (Girard et al., 2021; Martinez et al., 2017). Research has shown that MLPs enhance care in two ways: first, they improve provider communication and collaboration, and help clinicians build relationships with other service providers (e.g., case managers) (Tobin-Tyler & Teitelbaum, 2019); and second, they improve outcomes by empowering providers to screen and refer for HHLN and other SDOH (e.g., case managers, patient navigators) (Davis et al., 2022) and reduce barriers to care (Tobin-Tyler & Teitelbaum, 2016). Additionally, MLPs have been shown to be a cost saving, effective model of care for patients and hospitals that invest in them (Connor, 2018). In this way, MLPs serve as an “upstream” intervention to address the SDOH before they can impact health and well-being and more importantly, as a way to bridge the gap between the healthcare and socio-legal needs of vulnerable populations (Tobin Tyler, 2019); as such, MLPs present a potential pathway for helping to address some of the heightened needs, or barriers to care, that women with co-occurring IPV/OUD have, and represent a promising opportunity for providers in terms of integrating care for this population.

To date, however, no studies have examined the potential benefit of using an MLP to help address co-occurring IPV/OUD. In fact, much of the research on MLPs has focused on the impact of these interventions with pediatric populations (Beck et al., 2012, 2019, 2022; Pettignano et al., 2013; Rosen Valverde et al., 2019; Ryan et al., 2012; Taylor et al., 2015; Weintraub et al., 2010). These studies have demonstrated the power of MLPs to improve the health and well-being of children and their families through resolving a variety of socio-legal needs, including access to benefits (e.g., SSI, Medicaid, SNAP, etc.), housing issues/landlord-tenant disputes, educational issues, utility shutoffs, food scarcity, and general resource scarcity, among other issues (Beck et al., 2012, 2019, 2022; Pettignano et al., 2013; Rosen Valverde et al., 2019; Ryan et al., 2012; Taylor et al., 2015; Weintraub et al., 2010). Other studies have focused on populations of veterans (Tsai et al., 2017b), homeless (Tsai et al., 2017a), immigrants (Fuller et al., 2020; Sauaia et al., 2022), cancer patients and/or other critically ill patients (Eynon et al., 2020; Rodabaugh et al., 2010), and general low-income populations (Cené et al., 2022; Hernández, 2016; Liaw et al., 2023; Sauaia et al., 2022), and likewise have shown significant results in terms of improving the health and wellbeing of these populations. Furthermore, several studies have also shown that not only are MLPs effective for addressing unmet needs among vulnerable populations, but they are also effective for teaching clinicians about the SDOH, and for empowering clinical staff to address SDOH-related needs amongst their patient populations (Beeson et al., 2013; Girard et al., 2020; Tobin-Tyler et al., 2014; Tobin-Tyler & Teitelbaum, 2016; Welch et al., 2021). However, while a few studies have included the provision of family law/domestic violence legal aid in their measures (Cené et al., 2022; Mapp et al., 2022; Pettignano et al., 2013), no MLP work—to our knowledge—has specifically sought to address IPV victims as a population of interest; likewise, there has been limited focus on individuals with SUDs (Chaudhary et al., 2018; Tsai et al., 2017b). Lastly, most MLPs have focused on the need to provide civil aid to those experiencing health HHLN; the extent to which such models can be used for assisting populations like women with co-IPV/OUD who have both civil *and* criminal defense needs has not been well studied (Vanjani et al., 2020). To that end, we conducted a qualitative interview with individuals whose work brings them in contact with women experiencing co-IPV/OUD to understand their perspectives on using an MLP to care for this population. This article specifically examines participants’ thoughts on the barriers and facilitators to employing an MLP in this context.

## Method

This article draws from data collected as a part of a larger qualitative project on improving service provision for women experiencing co-IPV/OUD. As a part of that study, we interviewed individuals (*N* = 40) working across service domains (e.g., substance use, IPV, healthcare, human services, criminal justice, social work, behavioral health, etc.) about their experiences working with women with co-IPV/OUD. The goal of the parent study was to understand the ways in which service providers currently care for and treat women with co-IPV/OUD, what barriers and facilitators for providing care for this population exist, and how services might be integrated to provide more coordinated or co-located care. We also sought to understand providers’ knowledge of MLPs, as well as their perspectives on the feasibility of developing such partnerships. This article uses the data from that study to present the findings on the barriers and facilitators to employing an MLP in settings which serve women with co-IPV/OUD.

Data collection for this study began in the fall of 2022 and ended early spring of 2023. Any service provider in the state of Pennsylvania whose work was related to IPV or OUD and/or brought them into contact with women experiencing IPV or OUD, including those individuals in IPV advocacy, substance use treatment, criminal justice (including law enforcement, institutional and community corrections, parole and probation, and problem-solving courts), healthcare provision, social work, human services, and academic or research settings were eligible to participate. Recruitment was carried out using a snowball sampling methodology. For the first wave of recruitment, the study team compiled a list of existing professional contacts from relevant fields and sent each of these individuals an initial recruitment email with an explanation of the study and how to participate. Second, we asked individuals from the first round of recruitment who completed the interview to assist us in identifying additional potential participants. Initial participants assisted us in two ways, either by providing information about the study to other eligible colleagues or by providing the contact information of other professionals in their networks who were working with women experiencing IPV, OUD, or both.

The interview guide asked participants to broadly describe their past or current experiences working with women experiencing IPV and OUD, what barriers they had encountered in their work, including any challenges that impact engagement in care and retention, any facilitators to treatment or ideas on how to effectively provide care for this population that were important, their thoughts on how to improve care and treatment for women with IPV and OUD, their knowledge of MLPs, any aspects of MLPs that were already represented in their agencies, and their thoughts on the feasibility of employing MLPs in treatment contexts where women with co-IPV/OUD were being served, including what barriers or facilitators might exist to doing so. Questions relevant to this analysis included, “What barriers do you think exist to developing medical-legal partnerships for women experiencing co-IPV/OUD?” and “What would help facilitate medical-legal partnerships for women experiencing co-IPV/OUD?” All interviews lasted between 60 and 90 min. As a thank you for participating in the study, providers were compensated with a $50 gift card or cash incentive. The Institutional Review Board at the Pennsylvania State University approved this study.

All interviews were conducted on Zoom and recorded and then transcribed by a trained transcriptionist with expertise in qualitative interviewing. The interview transcripts were then reviewed for accuracy and revised, if needed, by a qualitatively trained research assistant, who then uploaded them into Atlas.ti for data management and analysis. Analysis took a two-coder iterative approach, focusing on content coding of themes and subthemes. In the first step, each of the two coders reviewed all transcripts line by line to identify initial themes and subthemes. The coders then met to review their work and consolidate themes into major and minor codes. The result of this process was a preliminary codebook with delineated exclusion and inclusion criteria for each code. In the second step, each of the coders re-coded all transcripts using the codebook and met a second time to review coding and reconcile any remaining differences. This process resulted in seven major codes related to MLPs, including the two presented in this study—“MLP barriers” and “MLP facilitators.”

After the initial coding, an Atlas.ti query was run to pull and review all quotes associated with the “MLP barriers” and “MLP facilitators.” Upon review of the data, it was apparent that each code encompassed several nuanced ideas that warranted further delineation into subthemes and thus, a second layer of analysis was conducted on these codes to describe each in more detail. First, the two co-coders each reviewed all components of the transcripts which had been coded as “MLP barriers” and “MLP facilitators” and made note of the emergent subthemes that arose in each. Next, the two coders met to compare their notes and consolidate them into two sets of subcodes, one for “MLP barriers” and one for “MLP facilitators.” The coders then independently reviewed all components of the transcripts coded with the codes again and applied the subcodes to each. In the last step, the coders met to compare all subcodes and reconcile any differences if needed. The results of this analysis are presented below. Selected quotes were those that accurately and faithfully captured the overall sentiments of most participants, were succinct, and provided the best, most illustrative example of each subcode, with consideration given to the representation of the diversity of participants in the sample. Additionally, we have included participant study identification numbers and organization types along with all quotes to highlight the perspectives as representing different service domains. A note on participant numbering: Participant numbers exceed 40 because we assigned numbers to participants as they were recruited. Thus, if a participant did not respond to our recruitment request, we did not use the number again.

A note of the presentation of the data: After completing the analysis, we recognized that the subcodes contained in the “MLP barriers” and “MLP facilitators” themes “mirrored” one another—or rather, represented “two sides of the same coin.” Rather than combine these two themes and present them as one, we opted instead to present each separately as (a) the questions utilized to elicit this information specifically asked about barriers and facilitators and thus, the responses presented are a reflection of participants’ thoughts on each of those topics independently; and (b) there are subtle nuances in each theme that we wished to capture and/or feared might be lost in a more broad presentation of these themes. Thus, to be true to our participants’ reflections on each and provide an authentic representation of the data, we present each theme separately.

## Results

### Sample Characteristics

Forty open-ended interviews were conducted with service providers whose work was related to IPV and OUD. Participants were mostly white (87%) and female (84%), from a diverse set of service domains, including social work/human services (20%), drug and alcohol treatment (20%), healthcare (15%), IPV advocacy (15%), criminal justice (12%), and behavioral health (10%). Participant titles and roles within their organizations varied; however, the most common positions within our sample included administration (25%), clinician (20%), social worker (15%), supervisor (13%), and probation officer (10%). Participants reported an average of seven and a half years at their current position (range 1–40 years).

### Barriers

#### Working Across Medical and Legal Systems

Participants felt that a potential barrier to developing MLPs for women with co-IPV/OUD would be getting the medical and legal systems to work together. Some described clinicians’ potential reluctance to work with lawyers, “The biggest barrier … clinicians don’t want to work with lawyers. Lawyers sue doctors [laugh]. You know being afraid of bringing lawyers into the clinic in any capacity because they think that is going to be threatening (7; criminal justice).” Others described the potential difficulty in getting lawyers to invest their time in MLPs:

Like the legal side, what is the benefit to them if they are volunteering their time? Is it because they care about this population or is it because it makes things easier for them when folks come through? I just feel they are busy and have a lot going on and I don’t know what would bring them to the table. Not just lawyers, but just the whole legal side of the partnership (24; healthcare).

Others more generally expressed what they saw as difficulty getting healthcare and socio-judicial systems to work together:

Just getting the systems to work together, like medical and legal systems sometimes don’t exactly mesh well. Just getting everyone who would be involved onboard…. Like making people understand why this is necessary and then getting them onboard with all the aspects of it (41; social work/human services).

Thus, convincing medical and legal professionals that an MLP was a positive, safe, and potentially mutually beneficial model of care was seen as a challenge. Another participant stated, “…someone from the legal side would need to understand the medical culture and vice versa…. I guess that could be a challenge, just figuring out how to intersect with the different cultures of the legal world and the medical world (40; social work/human services).”

#### Silos

Relatedly, participants also described the issue of working in “silos”; or rather how sometimes service providers who work with women experiencing co-IPV/OUD do not interact much with each other. As one participant stated:

A lot of service providers don’t talk to other service providers, like we don’t have a relationship with housing service providers. Even the providers that interface with this population and know they are experiencing challenges related to housing … they aren’t having those conversations with those who are providing those services. Different systems are often very reluctant to talk to each other, to connect, share best practices, I don’t see those conversations happening…. I think that is a challenge (8; healthcare).

Another similarly stated, “Everyone walks around in a silo … focus[ed] on their thing…. The barrier is how do we come with our baskets of stuff and figure out the one thing we can do as a group to help a survivor (21; IPV advocacy).” Participants felt that often providers from different disciplinary backgrounds have different ideas about what the best approach to addressing a problem like co-IPV/OUD might be:

There are cultural issues that can be hard in terms of how people do their jobs or how they approach a problem; helping people shift toward thinking more about what are we trying to achieve, what are our shared goals, as opposed to oh I just must do this task, right (7; criminal justice)?

Additionally, different providers have different ideas about what the outcome should be for their patients/clients with IPV and OUD:

Legal aid organizations … are used to thinking about success as I won this case … [so] getting them to think more holistically about the value of legal support can be hard. So, in a medical-legal partnership we don’t count success as just a legal win in court. We count it as how we are supporting this patient in multiple ways (18; criminal justice).

Thus, overcoming disciplinary silos and finding common ground and/or common objectives for working with women with co-IPV/OUD was seen as a barrier to MLPs. As another participant stated, “Everybody is walking in with their own goal, like probation, they got a goal they are working towards. It [would] take a year or two of really working together before you could do what you are going to do (23; drug and alcohol treatment).”

#### Staffing

Another barrier endorsed by our participants was staffing. Participants expressed that their agencies were already suffering from a staffing shortage. As one participant stated, “Staffing is hard. Staffing is very hard. Everyone is understaffed and anytime there is a staffing concern, that runs into anything (32; drug and alcohol treatment).” This was true for both participants who worked in healthcare and the socio-judicial system. As one healthcare worker stated, “We don’t have enough GPs (general practitioners) by a long shot. To try and get trained physicians assigned to you know drug courts and following up with some of these people, it would be quite a reach (34; healthcare).” Similarly, an individual who worked in the socio-judicial system stated, “It is really tough to sort these things out, when we certainly don’t have the personnel to do justice to each one of these situations (18; criminal justice).” A lack of staffing in general, therefore, was seen as a barrier to implementing MLPs.

Participants also discussed the difficulties related to finding staff that could support an MLP in their agency. For some, implementing MLPs meant hiring new staff. However, getting their agency to approve a new position was a challenge. As one participant stated, “There's so many barriers to push a new position, that would be our biggest issue, there's like three giant levels we would have to go through so you can get something like that (11; social work/human services).” Others, however, stated that even if their agency approved of a new position, finding someone to hire was a challenge. As one stated, “For our facility, one of the barriers we see is staffing, even if we created a position, finding someone to fill it (31; behavioral health).” Participants expressed two hurdles in terms of hiring support for the MLP. The first was a lack of competitive wages; as one participant stated, “Wages are very low. So, it is hard to recruit. Low wages make it hard to recruit additional people… (34; healthcare).” The second was finding individuals who were appropriately trained in IPV, OUD, and related issues (e.g., crisis management). As one participant stated:

Having enough people to be able to provide those services who are trained. There is so much turnover everywhere, like how you would ensure that people were trained to complete that type of work … IPV, drug counseling, crisis counseling training – [it] takes a lot of time to be able to complete that training. So, making sure someone is trained to do that type of thing and keeping that staff on board to be able to ensure consistent services I think is a challenge (41; social work/human services).

Another similarly stated, “Having people who are educated in this specific issue, who are trauma informed, who know how to have these conversations, and then who are willing to have them not for a couple of months, but on a continued basis (45; IPV advocacy).” Thus, finding and keeping individuals who were trained in areas relevant to MLPs was also seen as a challenge.

#### Funding

Lastly, and perhaps unsurprisingly, funding was another barrier to implementing MLPs for women with co-IPV/OUD. Participants recognized that funding would be needed to sustain the program and pay clinicians and legal personnel for their time:

I think another barrier would be on the end of the providers too, like how long would they feel comfortable volunteering or doing pro bono work for before they want to be compensated or how long could that model be sustainable for before additional funding or grants would be needed to keep that kind of program going (35; behavioral health).

Another participant similarly stated, “You have to find the money to pay everybody. The legal advocates, the attorneys, secretary, paralegal. Everyone needs [sic] paid. It is not volunteer work unfortunately; attorneys are not handing their time over for free (44; criminal justice).” However, where funding would come from for such a program was unclear. As one participant stated, “Funding for something like that, where does the money come from? That is always the issue (5; drug and alcohol treatment).” Another offered, “Funding because that is *always* a challenge, right? Who funds what agency and if you don’t have the funding for it or the hospital doesn’t want to pay for that, that is the biggest barrier that I can see (12; social work/human services).” Thus, participants felt like even if they could bring together the medical and legal sides to work on an MLP, finding sustained funding for the program would be a challenge. As one participant stated, “I think sustainability and funding is always a challenge, maybe I have great buy in from the health and legal sides, and everyone is excited but there is not a sustainable funding model and so that is challenging (7; criminal justice).”

Participants also stated, however, that even when funding was available, there were often restrictions or constraints placed upon its use—constraints that might prohibit their agency from using grant monies for the purpose of an MLP. One stated, “We have a lot of structural barriers to how [our] money is utilized. Like the decision-makers, the bureaucracy involved. There are always some strings attached in some way, right? So how would that work (20; healthcare)?” Funding constraints limited agencies’ ability to utilize grants towards new initiatives or other programs that may not have immediately quantifiable outcomes. As another participant stated, “It takes a lot of work to find funders, and, for example, our agency sometimes will focus on different funders. They set the restrictions and will say maybe you have to take X number of cases a year (44; criminal justice).” As such, agencies might be reluctant to engage in activities that may upset their funders:

I think another barrier, how certain funders will interpret partnerships that don’t seem aligned with that funding source. If you are funded by one of your big donors you don’t want to jeopardize that relationship, maybe you are going to be like well, they are a funder, they give us some money. We get stuff from them. There is not a lot of opportunity to speak up and push back (21; IPV advocacy).

Thus, finding funding that could be utilized for an MLPs and that would help to set it up and sustain it was also seen as a barrier. Another participant stated, “Money talks. Any kind of funding to get it started, to have it work. But then also is it going to be a free service, or will the cost come down to the individual? So, paying ongoing like that (32; drug and alcohol treatment).”

### Facilitators

#### Buy-in

Participants felt that for an MLP for women with co-IPV/OUD to be successful, it would need “buy-in” from key stakeholders, including patients/clients, service providers, and administrators. First, patients/clients would need to be willing to use the MLP and its services. As one participant stated:

I mean I think a huge part of it would be the trust involved. Or perceived trustworthiness, because if people are not connecting and aren’t hearing each other or lecturing to each other, not much progress will be made. You can get great advice but if you don’t trust the person or like the person it might just stop there (47; healthcare).

Likewise, another stated, “I think first like buy in. Make sure people know that they are there and getting people to utilize them (41; social work/human services).” Second, providers would need to believe in the work of the MLP and see the value of such a partnership. As one participant stated, “Having champions… People that are really bought in, whether it is the lawyer who feels like having that collaboration with the clinicians or the clinical staff helps them do their job better or vice versa (20; healthcare).” Another similarly offered, “You would need to get the necessary healthcare providers and people who you would need to be involved onboard … to figure out how everybody is going contribute (35; behavioral health).” Thus, providers from both the medical and legal contexts would need to “buy-in” to the MLP and be willing to do the work:

Making sure that you have the right people in the right roles because what can happen sometimes is … you set up a partnership [but] … you don’t have the right clinicians who are really committed to the model doing the daily work or vice versa and I think that is true with the legal side as well. Spending the time to make sure people are bought into this more holistic model (9; criminal justice).

Third, administrators would likewise have to see the value of an MLP and support providers in their ability to engage in such partnerships. As one participant stated:

You can have wonderful people at the clinical level and the legal level who are bought in, who want to do this. But if you don’t have systemic support, administration, or directors on board, then the staff are not going to be able to change their practice in a way that is going to support the partnership. I think that is critically important (6; social work/human services).

Another participant stated, “We can advocate and do as much as we can, but without sign off it's hard to do anything. It's hard to get anything positive started (18; criminal justice).” Administrative support, therefore, was seen as vital to ensuring the longevity and success of an MLP. As another participant stated, “If you have the administrative buy in, like truly have the administrative buy in, you are going to get the funding. If somebody at the top is committed to making it happen, it is going to happen (45; IPV advocacy).” Thus, both service providers and administrators needed to be onboard in order for an MLP to be successful:

We will see people on the ground really invested and not have the support of the administrative folks. We have also seen administrative folks who are like this is a great idea. We want to do this, but they don’t get the investment of the people who are supposed to be doing the work on a daily basis. So you have to have both for it to work (7; criminal justice).

Lastly, some participants felt there also needed to be “buy-in” from other stakeholders, namely funders and policy makers. For example, one participant stated, “[Service organization] is pretty open towards … doing what they can to provide services in-house. [But] we get a little pushback from our payers, insurance companies … so getting buy in from the powers that be [funders] (43; drug and alcohol treatment).” Having funders who saw the importance of the MLP and what it could provide was therefore important; another participant stated, “We probably need some funding too. Like a board of rich donors, who are bought into the model (20; healthcare).” Other participants discussed the need for policy makers to “buy-in” to MLPs as a model of care. As one participant, when discussing the need for policy maker “buy-in,” stated, “[You would need] probably several who would see the benefit in this and would see how important it is and be happy to put their time into this. It would take really thinking about the societal impacts and downstream benefits (22; behavioral health).” Thus, to make real changes and implement policies that support holistic models, like MLPS, policymakers needed to be on board and see the value in such partnerships. Another participant stated, “There would have to be buy-in from at least from the local leadership, right? Local politicians, whether that is going to be based on like the county or that is the state. Definitely getting the buy-in with that (39; behavioral health).”

#### Collaboration

Providers also described establishing meaningful collaboration across service provision domains as something that would be needed to successfully employ an MLP model. Different providers had different ideas about how collaboration could best be achieved. Some providers felt this kind of collaboration would require individuals providing MLP services to have an “open mind.” As one participant stated, “Open-mindedness. Being able to have this mentality right, that just because we have always done it this way, doesn’t mean that is the only way and doesn’t mean that is what works best (26; healthcare).” Thus, getting providers to think differently about the work that they do would be necessary. Another participant stated, “Like community mindset as opposed to individualistic mindset. Shift into that mindset and know this isn’t like a dig on your ego… We would have to be thinking more of the community setting and the community good (39; behavioral health).” Some providers felt that relationship building was key to collaboration. As one participant stated, “[What] would assist it? Relationships, relationships, relationships. Just knowing the people involved and having sound relationships with them. I always felt like the relationships I make with the people that are involved with my client, aid the client (33; drug and alcohol treatment).” Another similarly stated:

The people involved; do they trust each other? I have faced different circumstances where different agencies are involved and don’t work well together and that absolutely has implications for the patient. Whether it is on a personal level, they don’t like each other, or they have been burned in the past or whatever it is. So, trustworthiness – basically how people get along (47; healthcare).

Thus, having strong relationships among providers and, in particular, relationships which were built on trust and which centered patients/clients and put their needs first was seen as an important facilitator for MLPs. Additionally, some participants felt that having a set of common goals across the medical and legal service domains was important for fostering collaboration. As one stated:

Having shared goals for this client/patient … that includes learning from each other so the clinicians understand how to think about when this might be a legal issue that needs support and the lawyers understanding the value of having a clinical partner to help them do their job, all of those things (26; healthcare).

Thus, having shared goals that were rooted in providers’ mutual understanding of each other and what each service domain brings to the partnership was important for fostering collaboration and for the success of an MLP. As one participant stated:

The facilitators are really when people sit down and before they even start trying to partner, don’t build a partnership and just jump in. You need to talk about why you are doing what you are doing. You need to understand each other's perspectives, understand what people do on a daily basis as lawyers and clinicians right? And then figure out how you are going to best build this system that is going to support your patients and clients with those goals in mind (7; criminal justice).

#### Training

Participants felt that more holistic training across the fields of IPV and OUD would also be required to help facilitate an MLP model. Participants felt that providers working with women experiencing IPV and/or OUD may not recognize how connected these issues truly are. As one participant stated,

People don’t realize the intersection between the two … I don’t know … if it is just the lack of education but there is so much of a connection … and in the beginning, you might not be able to connect it and so having that education. A lot of staff that are in that field – well even some staff here, we train staff but not specifically on all these issues. I keep saying this, the connection between the two I think so that education piece which I already mentioned before (17; social work/human services).

Participants felt, in general, therefore, that more training was needed across service provision domains on IPV, OUD, and the intersectionality of the two for MLPs to be successful. As one participant stated:

I’ve been in this agency for almost 22 years now and you do not see training specific to substance abuse and interpersonal violence. They might touch on it here and there. But those are really few and far between. I think anybody who is working in this [MLPs] needs to be trained on this (36; drug and alcohol treatment).

Another participant similarly stated, “You know so some sort of training, you know whether it be to medical students or some sort of seminar sort of demonstrating the impact, the potential impact of these sorts of interventions (8; healthcare).” Participants also felt, however, that individuals working in an MLP context would need additional training in the processes that occur in both the medial and legal settings for the partnership to be successful. As one participant stated:

Well, I think there should be some cross-education, what is the process, what documentation is necessary. How can I be helpful in my documentation? I’m sure they [lawyers] would like to understand what is important to help somebody maintain their recovery (20; healthcare).

Another participant stated, “Training everybody to make sure they understand what the program is, what it is not, when to contact the team or person, expectations for follow-up. When should a referral be made? How is screening done? So, training is really important (43; drug and alcohol treatment).” Thus, training on the intersectionality of IPV and OUD, as well as on how the medical and legal systems work would be necessary for providers involved in an MLP.

#### Dedicated Personnel

Lastly, participants also felt that having a dedicated person, whose sole role was to serve the MLP, would help to facilitate the partnership. As one participant stated, “What do we need to do in order to do that? Have people on board for stuff like that (25; drug and alcohol treatment).” Another similarly stated, “It is a little bigger than two individuals. You need a person who, that is a part of their job and what they need to do. It is not just extra work they have to do now (17; social work/human services).” Thus, participants felt that having someone on staff within the agency where the MLP was being employed who could focus solely on facilitating the partnership would be beneficial. Another participant stated:

If there could be a particular person that was staff to focus on this issue you know because this could be a full-time position for many. You still would do the direct service work but part of the position would be to work on that systems work where you are focusing on how can we make these changes and getting everyone at the table. So, that is their main focus for that role. They can’t say I don’t have the time because that is part of the position (32; drug and alcohol treatment).

## Discussion

We conducted semistructured, open-ended interviews with providers working across service domains to understand their perspectives on the barriers and facilitators to employing a medical-legal partnership for women with co-IPV/OUD. Interestingly, all but one of the themes that emerged from participants’ discussions of potential barriers and facilitators “mirrored” one another—or rather, represented “two sides of the same coin.” Thus, our key findings highlight some considerations that may need to be addressed before developing an MLP in this context—namely, the need for “buy-in” from all stakeholders, the need to establish shared goals, objectives, and practices across stakeholders, the need for training to ensure that all stakeholders have a holistic view of IPV and OUD, and/or the need for additional staff to facilitate the MLP. Furthermore, our study finds, as have many others, that significant investments in co-located or coordinated care for women with co-IPV/OUD are needed. Several of these themes have been identified in previous studies addressing barriers and facilitators to the integration of evidence-based substance use services in primary care settings, or the adaptation of evidence-based substance use treatment for vulnerable populations; for example, inadequate (or inadequately-trained) staffing, uneven buy-in, and insufficient funding are well-known barriers to developing other innovative models of care, while the recruitment of dedicated personnel and a collaborative mindset among stakeholders have emerged as key facilitators (e.g., Adeniran et al., 2023; Hirchak et al., 2023; Lash et al., 2011). At the same time, this study found unique impediments—and opportunities—for the creation of MLPs, namely the difficulties in working across complex medical and legal systems, a barrier perhaps best addressed by “MLP champions” both within and outside the organizations involved.

We found that participants cited difficulties working across the medical and legal systems as a potential barrier; at the same time, they also endorsed “buy in” from all stakeholders as a facilitator—including not only from medical and legal professionals, administrators, and policymakers but also from patients/clients. Studies of MLPs have recognized some of the pitfalls of working across the medical and legal systems and highlighted some of the procedural, ethical, and other hurdles that must be overcome, including the need to demonstrate the value of such models to both clinicians and legal personnel (Mantel & Fowler, 2020; Mantel & Knake, 2018; Newman, 2012). Thus, the idea that clinicians and legal personnel need to “buy in” to an MLP for it to be successful is not surprising; however, studies show that, in general, despite systemic differences in medicine and law, working across these two systems has not been an impediment to such partnerships and in fact, MLPs have only served to increase clinicians’ and legal personnel's willingness to collaborate with one another (Boumil et al., 2010; Mantel & Fowler, 2020; Tobin-Tyler & Teitelbaum, 2019; Wettach, 2007). We will return to a discussion of this point shortly; however, what is more interesting, and perhaps where some consideration and thought may need to be directed, is regarding the issue of “buy in” from the patients/clients—especially when considering the complexity of socio-legal needs that women with co-IPV/OUD might have. Traditionally, MLPs have primarily sought to provide civil aid to those experiencing HHLNs, with limited emphasis on criminal defense (Vanjani et al., 2020). Women experiencing co-IPV/OUD admittedly may have both civil AND criminal legal concerns—and as stated earlier in the introduction, their criminal concerns may prohibit them from seeking and receiving care out of fear of the socio-judicial consequences that may result from doing so (Morrison et al., 2022; Phillips et al., 2021). Thus, providers wishing to introduce an MLP model of care for women with co-IPV/OUD may want to consider expanding the partnership to include public defense—while doing so may add a layer of complexity to establishing such a partnership, it may also reduce barriers to disclosure among women with co-IPV/OUD and increase their willingness to seek help if they know they will receive assistance for their criminal legal concerns, as well as their civil ones.

Next, we found that participants felt that disciplinary silos—or rather, differences in providers’ priorities, approach, and desired outcomes—was a barrier to employing an MLP for women with co-IPV/OUD and stated that in order for such partnerships to work in this context, stakeholders would need to develop collaborations that were patient/client-centered, and built on trust and mutually agreed upon sets of practices and goals for patients/clients. As stated earlier, despite the systemic differences that exist with the medical and legal systems, research shows that MLPs *have* been successful at fostering interdisciplinary collaboration and cross-disciplinary communication, especially for medical providers (Beeson et al., 2013; Tobin-Tyler & Teitelbaum, 2019; Wettach, 2007). Research on MLPs has found that incorporating these partnerships into clinical settings helps increase providers’ receptiveness to, and comfort with, engaging in screening and referral for HHLNs and other SDOHs, and enhances their work with other providers (e.g., case managers, patient navigators) (Beeson et al., 2013; Davis et al., 2022; Murillo et al., 2022; Murphy et al., 2015; Tobin-Tyler & Teitelbaum, 2016). MLPs also benefit clinicians and lawyers by clarifying each provider's role in care and increasing their ability to reduce barriers to care for their patients/clients (Boumil et al., 2010; Tobin-Tyler & Teitelbaum, 2016; Wettach, 2007). MLPs, therefore, have a demonstrated record of empowering providers to engage in more cross-disciplinary, collaborative action on behalf of their patients/clients (Tobin-Tyler & Teitelbaum, 2016). However, these partnerships have typically been housed in hospitals or community clinic settings and largely have included physicians from family medicine, primary care, or other more generalized settings (Tobin Tyler, 2019; Tobin-Tyler & Teitelbaum, 2019; Wettach, 2007). Furthermore, research on women experiencing IPV and/or OUD shows that this population may seek help from a wide range of providers and/or may utilize providers outside of traditional hospital or community clinic settings (e.g., medication-assisted treatment providers [MAT], or harm reduction organizations such as syringe exchange programs) (Mason & O’Rinn, 2014). Thus, providers wishing to develop an MLP for this population may want to consider developing partnerships that stray outside their established networks and intentionally include a heterogeneous group of service settings. While this may present a time-intensive challenge for providers with many competing priorities, such a strategy may yield better buy-in, and coordinated care, for women with co-IPV/OUD.

We also found that participants saw staffing as a barrier and most believed that to be successful in this context, MLPs might need to have a dedicated staff member who could facilitate the partnership. Additionally, participants also felt that all stakeholders involved in the MLP would need training in the unique aspects of IPV and OUD. Staffing can be a barrier for MLPs and the notion that additional support for physicians and legal personnel involved may be needed has been explored elsewhere in the literature (Colvin et al., 2012; Tsai et al., 2017a). However, one study by Colvin et al. (2012) has argued for the inclusion of social workers into the MLP model. They argue that social workers are primed to effectively “bridge the gap” between the medical and legal worlds and can enhance MLPs by bringing a more systems-level approach to resolving HHLNs. Social workers take a family systems approach and may have an ongoing relationship with patients/clients in ways that neither physicians nor legal personnel do—thus, they can assist MLPs by identifying the root causes of HHLN and other SDOH-related health issues and help physicians and attorneys seek more long-term, permanent solutions (Colvin et al., 2012). Additionally, social workers are employed in most hospitals or community health clinics and in other settings where women with co-IPV/OUD are seen (U.S. Bureau of Labor Statistics, 2024). Thus, including social workers in MLPs for women with co-IPV/OUD may be a viable way of providing additional staff support for the partnership—it may also be a way to address some barriers regarding a lack of training around IPV and OUD. Research shows that when clinical curricula include MLP training, clinicians and other medical providers gain significant increases in their understanding of the SDOH that impacts health (Anderson-Carpenter et al., 2013; Teitelbaum & Lawton, 2017; Tobin Tyler, 2012, 2019; Tobin-Tyler et al., 2014; Tobin-Tyler & Teitelbaum, 2016, 2019). Theoretically then, providers who have experienced MLP training should have a good understanding of the roles that social issues such as IPV and substance use play in their patients’ health outcomes. In the short term, however, the inclusion of social workers within MLPs may bridge extant training gaps, given that social workers are well-equipped to assess all of a patient/clients’ psychosocial health needs; they may also help clinical and legal staff navigate their work from a more holistic perspective. Another potential avenue for bridging the gap between the medical and legal spheres of an MLP might be the inclusion of certified peer recovery specialists or patients who have lived experience of co-IPV/OUD and who can help others navigate the complexities of the socio-judicial and healthcare systems. More research, however, on the implementation of MLPs and what best practices might serve these partnerships in the care of women with co-IPV/OUD is needed.

Lastly, participants identified funding as a potential barrier to employing an MLP in this context. This is not surprising, and research on *both* MLPs and co-occurring IPV/OUD have cited funding constraints as a significant impediment to providing integrated care for patients (Morrison et al., 2022; Regenstein et al., 2018; Sandel et al., 2010; Stone & Rothman, 2019; Tsai et al., 2017a). However, this is one area in which MLPs may also prove to be most beneficial—MLPs, in fact, have demonstrated compelling results in terms of their ability to be economically viable for both clinical systems and patients. Studies have shown that MLPs have been cost saving for hospitals who have invested in them and have helped providers and hospitals recover funds previously denied by Medicaid and Social Security (Atkins et al., 2014; Beeson et al., 2013; Connor, 2018). They have also helped to reduce hospital admission and readmission rates and visits to the emergency room, increased access to benefits and insurance, and ensured reimbursement or compensation for healthcare dollars spent—all outcomes that financially benefit both clinics and patients/clients (Beeson et al., 2013; Benfer et al., 2018; Cené et al., 2022; Eynon et al., 2020; Hernández, 2016; James et al., 2020; R. Pettignano et al., 2014; Regenstein et al., 2018; Sauaia et al., 2022; Weintraub et al., 2010). Thus, MLPs represent a potentially cost-effective way to provide more integrated and holistic care for women experiencing co-IPV/OUD and thus, may be an in-road into providing the kinds of co-located or coordinated care for this population highlighted in the literature (Morrison et al., 2022; Stone & Rothman, 2019). Future research should focus on the cost-saving potential of MLPs in caring for populations of women experiencing IPV and/or OUD to identify the financial benefits of using such a model. Such research could be used as well to demonstrate to funders, hospital administration, and other relevant stakeholders the advantages of investing in MLPs for their patients/clients.

### Limitations

There are several limitations to this study that need to be mentioned. First, while we strove to include a diverse set of providers in our sample, some service domains were underrepresented. Specifically, our study recruited more providers working in health care and adjacent fields (such as drug and alcohol treatment), while individuals in the criminal-legal sectors were fewer in number; in turn, our findings might be biased toward the experiences of the former group of professionals. Additionally, all participants were recruited from Pennsylvania, with a good majority working in the western portion of the state and/or within the greater Pittsburgh region. Thus, our findings may not be generalizable to providers working in different states and regions of the United States. Moreover, recent estimates indicate that the MLP model is more prevalent within the northeastern United States. It is thus possible that more numerous, or different, barriers to MLP establishment exist within other regions of the country (National Center for Medical-Legal Partnership, 2024). This article also focused broadly on the barriers and facilitators to employing MLPs in care contexts for women with co-IPV/OUD; thus, some of the subtle nuances of care (e.g., provider vs. patient priorities, provider differences in treatment approach) and the challenges they present in terms of providing patient-centered care in the context of an MLP were not within the scope of the work. The study is therefore limited in terms of the recommendations it can provide for addressing these nuances. Additionally, our findings may be limited in terms of their relevance to other types of SUDs. Admittedly, many of the barriers to care-seeking that women with OUD face are prevalent among other populations of women experiencing different forms of substance abuse (e.g., stimulant use disorder, etc.); however, given that we focused on OUD, it is unclear to what extent our results are relevant to the barriers and facilitators that may exist for employing an MLP in context to co-occurring IPV and substance use more broadly. Finally, providers in our sample reported on average relatively long tenures within positions often characterized by high turnover. In this way, their views and experiences may depart from professionals in these fields at large. In the future, research in this area should seek to incorporate a more balanced sample of providers whose work brings them into contact with women experiencing co-IPV/OUD while expanding states and regions (such as the South, West, and Midwest) where MLPs are less established.

### Conclusion

We conducted open-ended interviews with providers across service domains to understand the potential utility of a medical-legal partnership for women experiencing co-IPV/OUD. As a part of that study, we explored some of the barriers and facilitators to implementing MLPs for this population and found that providers described a set of concerns around provider “buy-in,” differing priorities or silos of care, staffing needs, and funding issues that need to be considered if an MLP is to be successful in this context. This study suggests that MLPs may indeed present a comprehensive and coordinated care model for women experiencing co-IPV/OUD. However, future research should consider the best practices of the implementation of MLPs, including exploring the nuances of care for women with co-IPV/OUD and the potential benefits and pitfalls of using an MLP to deliver patient-centered care for this population, as well as the logistical considerations regarding who should be included in such partnerships, where partnerships should be located, and how effective, both from a cost-saving and outcomes perspective, such partnerships are as compared to other models of care.
